# Characterization of plant growth promoting activities of indigenous bacteria of phosphate mine wastes, a first step toward revegetation

**DOI:** 10.3389/fmicb.2022.1026991

**Published:** 2022-12-15

**Authors:** Najoua Mghazli, Odile Bruneel, Rahma Zouagui, Rachid Hakkou, Laila Sbabou

**Affiliations:** ^1^Center of Research Plants and Microbial Biotechnologies, Biodiversity and Environment, Team of Microbiology and Molecular Biology, Faculty of Sciences, Mohammed V University in Rabat, Rabat, Morocco; ^2^HSM, University of Montpellier, CNRS, IRD, Montpellier, France; ^3^Laboratoire des Matériaux Innovants, Energie et Développement Durable (IMED)_Laboratory, Faculty of Science and Technology, Cadi Ayyad University, Marrakesh, Morocco; ^4^Geology & Sustainable Mining Institute (GSMI), Mohammed VI Polytechnic University (UM6P), Ben Guerir, Morocco

**Keywords:** phosphate waste rocks, semi-arid climate, PGPB, revegetation, bioremediation

## Abstract

Morocco holds the vast majority of the world’s phosphate reserves, but due to the processes involved in extracting and commercializing these reserves, large quantities of de-structured, nutritionally deficient mine phosphate wastes are produced each year. In a semi-arid climate, these wastes severely hamper plant growth and development leading to huge unvegetated areas. Soil indigenous Plant Growth-Promoting Bacteria (PGPB) play a pivotal role in restauration of these phosphate mining wastes by revegetation, by increasing plants development, soil functioning, and nutrient cycling. The development of a vegetative cover above the degraded phosphate wastes, could stabilize and reintegrate these wastes in the surrounding environment. The current study’s objectives were to isolate, characterize, and identify indigenous bacterial strains, and test their PGP activity *in vitro* and, for the best-performing strains *in planta*, in order to assess their potential for acting as biofertilizers. A quantitative test for the synthesis of auxin and the production of siderophores as well as a qualitative test for the solubilization of phosphate were performed on all isolated bacterial strains. The production of hydrogen cyanide (HCN), exopolysaccharides (EPS), and enzymes were also examined. Three bacteria, selected among the best PGPB of this study, were tested *in planta* to determine whether such indigenous bacteria could aid plant growth in this de-structured and nutrient-poor mining soil. Using 16S rRNA gene sequencing, 41 bacterial strains were isolated and 11 genera were identified: *Acinetobacter, Agrococcus, Bacillus, Brevibacterium, Microbacterium, Neobacillus, Paenibacillus, Peribacillus, Pseudarthrobacter, Stenotrophomonas*, and *Raoultella*. Among the three best performing bacteria (related to *Bacillus paramycoides, Brevibacterium anseongense*, and *Stenotrophomonas rhizophila*), only *Stenotrophomonas rhizophila* and *Brevibacterium anseongense* were able to significantly enhance *Lupinus albus* L. growth. The best inoculation results were obtained using the strain related to *Stenotrophomonas rhizophila*, improving the plant’s root dry weight and chlorophyll content. This is also, to our knowledge, the first study to show a PGP activity of *Brevibacterium anseongense*.

## Introduction

Phosphate mining is one of the most important sectors of the Moroccan economy. Morocco has around 50 billion metric tons of rock phosphate reserves making it by far, the country with the largest reserves, and the 2nd biggest phosphate producer in the world, with annual production reaching 37 million metric tons in 2020 (USGS, 2020). During open-pit mining, huge volumes of wastes (hundreds of millions of tons per year) are removed to access the phosphate rocks. Wastes result also from the long process to generate the final product, implicating crushing, screening, and washing of phosphate rocks, followed by attacks with chemicals. All these represent approximately 18,000 tons of wastes every day, stockpiled on huge surface areas and exposed to atmospheric phenomena (Trifi et al., 2020; Mobaligh et al., 2021). These unstructured wastes, poor in nutrients, lacking organic matter, and contaminated by excessive phosphorus and sometimes hazardous contaminants such as fluoride, strontium, chromium, or cadmium, strongly limit the natural formation of a vegetative cover. These unvegetated wastes, damaging the esthetics of the landscape, are subjected to erosion and transport of small particles by wind and water and represent a potential threat to the environment and the local population’s health (Zine et al., 2020, 2021). The semi-arid climate, characterized by strong winds and short but intense rainfall events (annual precipitation not exceeding 250 mm), makes the establishment of a vegetative cover even more challenging. Furthermore, the salt concentrations at the surface of soils are generally high in semi-arid area due to low water infiltration and high evaporation rate (Mendez and Maier, 2008). Revegetation of phosphate wastes represents an effective and economic way to stabilize the ground, limit erosion, and allow the reintegration of the site in the surrounding ecosystem.

Due to their major role in soil functioning and plant development, indigenous plant growth-promoting bacteria could be of great help (Hassani et al., 2018). Numerous studies have been conducted on the use of indigenous bacteria to remediate contaminated sites (Wu et al., 2019; Jalali et al., 2020; Khairina et al., 2021). These bacteria act either through direct or indirect mechanisms (Mahmud et al., 2021). The direct mechanisms englobe (i) biological phytohormones production such as IAA (indole 3-acetic-acid) which plays an important role in root development and root hair formation, and hence in plant growth (Duca et al., 2014); (ii) some nutrients solubilization such as phosphorus or siderophores production important for iron captation (Upadhyay and Chauhan, 2022). Iron is a fundamental cofactor in cell machinery (Ma et al., 2016), and is highly abundant in soils but generally unavailable to microorganisms and plants. Siderophores are secondary metabolites that bind to iron and form an assimilable complex that provides plants with iron (Gray and Smith, 2005). Microbial siderophores usually have a higher affinity for iron than phytosiderophores (Pattnaik et al., 2019). The indirect mechanisms include the production of biocontrol agents such as hydrogen cyanide (HCN) which is known for its ability to prevent the development of pathogens; enzymes like cellulase, which can play an important role in restoring damaged soils by boosting seed germination or enhancing root development (Kuhad et al., 2011); and catalase which enhances plant defense against reactive oxygen species by decomposing hydrogen peroxide at an exceptionally rapid rate (Sharma and Ahmad, 2014; Nandi et al., 2017; Upadhyay and Chauhan, 2022). Exopolysaccharides (EPS) are metabolites produced by bacteria to protect themselves from harsh environmental conditions such as metals, drought or salinity (by sequestrating metals, or binding cations like Na^+^ alleviating salt stress, or enabling water conservation by maintaining a hydrated microenvironment around the cells) (Naseem and Bano, 2014; Sandhya and Ali, 2015). The EPS can also increase soil aggregation being adsorbed by some particles such as clay thus forming micro- and macroaggregates (Sandhya and Ali, 2015). Bacteria tolerating high concentrations of salinity can also be employed to counteract the negative effects of salt stress on plants, by enhancing physiological functions thus facilitating the installation and development of plants (Sharma et al., 2021).

To help establish a vegetation cover on these phosphate wastes, indigenous bacteria could represent an economical and eco-friendly method, acting as biofertilizer due to the properties described above. However, the documentation on plant growth promoting activities of indigenous bacteria from phosphate mine waste is poor. Therefore, the specific objectives of this study were to isolate and identify indigenous bacterial strains from phosphate mine wastes and characterize their PGP activities *in vitro* and for the best performing bacteria, *in planta*, in order to assess their use as biofertilizers. Three bacterial strains were selected for further biocontrol and metal tolerance tests and were used in single and consortium inoculation to evaluate their effect on the *Lupinus albus* L. growth, thus validating the results of *in vitro* PGP activities tests. This legume is an indigenous hyper-accumulator plant known for its capacity to adapt to harsh conditions such as drought stress, poor nutrients, and toxic metal contaminated soil, especially chromium, manganese, mercury, and lead-contaminated soils (Gawronski et al., 2011). This plant can also thrive on soils with very limited nutrient availability and a variability of pH from acid to alkaline (Esteban et al., 2003). *Lupinus albus* L. is also valued for its ability to improve soils by extracting toxic metals (Garau et al., 2021; González et al., 2021). The choice of *Lupinus albus* L. was based on (i) its ecophysiological traits allowing this indigenous legume to easily adapt to contaminated phosphate mining soils in Morocco, to (ii) its robust root system that facilitate the absorption of soil components (González et al., 2021), and to (iii) its ability to help recover de-structured soils *via* its capacity to provide nitrogen (Esteban et al., 2003).

## Materials and methods

### Experimental site and phosphate waste sampling

Samples of phosphate tailings were collected in October 2017 from one of the experimental cells established at the abandoned pyrrhotite Kettara mine near Marrakech in Morocco (31.8748, –8.1760). A rehabilitation scenario for this mine includes covering acidic tailings with alkaline phosphate mine wastes to limit water infiltration and hence acid mine drainage (Ouakibi et al., 2013; Bossé et al., 2015). Five subsamples were collected from different random locations, then were processed in the laboratory within a week of sampling. The physicochemical properties of these phosphate tailings were identified and are detailed in Mghazli et al. (2021) and Supplementary Table 1.

### Bacterial isolation

A soil solution was prepared using 10 g of phosphate tailings that were dissolved in 90 ml of sterilized physiological water (0.9% NaCl) and stirred for 1 h. A series of 10^–1^ to 10^–5^ dilutions were made from the soil solution, and 100 μL aliquots from each dilution were spread on a modified yeast starch agar medium (YSA) from Kadmiri et al. (2010). The modified-YSA medium contains glucose and starch as source of carbon instead of only starch, and it contains per liter 4 g of yeast extract, 2 g of starch, 8 g of glucose, 0.5 g of MgSO_4_ 7H_2_O, 0.5 g K_2_HPO_4_, and 15 g of agar. To select the maximum culturable bacteria, four different pH media ranging from 6 to 9 were used. The bacterial strains were transferred many times until a pure isolate was obtained.

### DNA extraction, 16S rRNA gene amplification and sequencing

**FIGURE 1:**
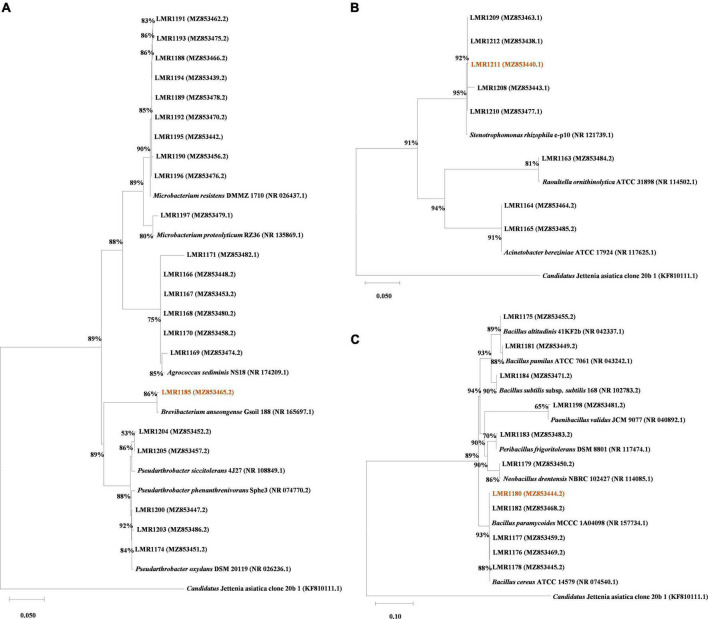
Phylogenetic trees of all isolated strains (indicated in bold) based on ClustalW aligned 16S rRNA gene. A total of 1,617 nt were used for *Actinobacteria* phylum alignment **(A)**, 1,526 nt were used for the *Proteobacteria* phylum alignment **(B)**, and 1,622 nt were used for the *Firmicutes* phylum alignment **(C)**. The Tamura-Nei model (Tamura and Nei, 1993) with a discrete Gamma distribution was used to construct the phylogenetic trees. Accession numbers are given between parenthesis. The bootstrapping replicates was 1,000 for all trees and the percentage of replicate trees is shown beside the branches. *Candidatus* Jettenia asiatica clone 20b_1 was used as outgroup, and the strains used for *in planta* test are highlighted.

Bacterial DNA was extracted using a standard phenol-chloroform protocol (Köchl et al., 2005), and 16S rRNA gene was amplified using MyTaq™ DNA Polymerase (Bioline, Meridian Bioscience, London, UK) and the universal primers 27f/1492r (Frank et al., 2008) or fD1/rD1 (Weisburg et al., 1991). The amplification was carried out as described in the following protocol: initial denaturation at 95°C for 1 min followed by 30 cycles of denaturation at 95°C for 15 s, annealing at 56°C for 15 s and extension at 72°C for 10 s, then a final extension step at 72°C for 1 min. The amplified products were subjected to Sanger paired-end sequencing and the resulting sequences were cleaned and processed using DNADragon and Mega X programs. The sequences were then BLASTed against the NCBI database to identify the isolated strains. All sequences were deposited in NCBI database and their accession numbers are shown in Figure 1 and Supplementary Table 2.

### Phylogenetic tree construction

The phylogenetic tree was constructed using Mega X (Kumar et al., 2018). All the isolated sequences were aligned alongside reference sequences from NCBI database and an external reference sequence [Candidatus Jettenia asiatica clone 20b_1 (KF810111.1)]. The Maximum Likelihood method was used. The evolutionary distances were computed using the Tamura-Nei model (Tamura and Nei, 1993) with a discrete Gamma distribution with 5 rates categories.

### Phenotypic characterization of the isolated strains

A pre-culture bacterial cell suspension was prepared in YSA medium and was used for all the following phenotypic tests. YSA medium with a pH ranging from 4 to 12 using KH_2_PO_4_/K_2_HPO_4_ and HCl/Tris buffer solutions, were used to study the ability of the isolated strain to tolerate different pH. While for the salinity test, YSA medium was supplemented with different concentrations of NaCl ranging from 3 to 9%. For these two tests, the bacteria were dot spotted in Petri dishes and incubated at 28°C for at least 48 h. The capacity of our strains to tolerate high temperatures was assessed in Petri dishes containing YSA medium incubated at different temperatures ranging from 35 to 60°C for at least 48 h. All tests were repeated three times.

### Plant growth-promoting traits

#### Auxin production

Pre-cultures were prepared and inoculated on a yeast mannitol medium (Sigma-Aldrich), supplemented with 0.5 g L-Tryptophane. After 48 h at 28°C, the cultures were centrifuged at 10,000 rpm for 20 min, then filtered through a 0.22 μm filter to eliminate any remaining bacterial cells. One mL of the filtrate was added to 1 mL of Salkowski reagent and incubated at room temperature in the dark for 30 min (Gordon and Weber, 1951). The optical density was then measured at 540 nm to quantify auxin production. The results were compared to a standard curve prepared using a pure IAA solution.

#### Siderophores production

A volume of the pre-cultures was inoculated on Luria–Bertani (LB) medium (Sigma-Aldrich). The tubes were incubated at 28°C for 7 days, then centrifuged at 10,000 rpm for 20 min and filtered through a 0.22 μm filter. The pH of the filtrate was then adjusted to neutral and 1 mL of the suspension was added to 1 mL of CAS reagent (Alexander and Zuberer, 1991) in hemolysis tubes pre-treated with HCl. The mixture was incubated at room temperature in the dark for 30 min, before optical density was measured at 630 nm. The semi-quantitative results were calculated based on the following equation:


$$\%SU=\frac{A\,r-A\,s}{A\,r}\times 100$$

Where %*SU*, % of siderophore unit; *Ar*, absorbance of the control; *As*, absorbance of the strains.

#### Phosphate solubilization

Ten microliters of the strain’s pre-culture were deposited in Pikovskaya’s medium (PVK) (Pikovskaya, 1948) containing tricalcium phosphate (TCP) as a source of phosphate. The Petri dishes were incubated at 28°C for at least 7 days. The presence of a transparent halo beside the colonies indicates TCP solubilization. The phosphate solubilization index (PSI) was calculated based on the following equation (Premono et al., 1996):


$$P\,S\,I=\frac{\left(C\,o\,l\,o\,n\,y\,d\,i\,a\,m\,e\,t\,e\,r+H\,a\,l\,o\,z\,o\,n\,e\right)}{C\,o\,l\,o\,n\,y\,d\,i\,a\,m\,e\,t\,e\,r}$$

#### Hydrogen cyanide production

The production of hydrogen cyanide (HCN) was determined using the protocol proposed by Castellano-Hinojosa and Bedmar (2017). For instance, bacterial strains were grown for 48 h in YSA medium supplemented with 0.4% glycine, then a sterile disc of 90 mm was dipped in 0.5% picric acid supplemented with 2% Na_2_CO_3_ and placed on the lid of each Petri dish, the plates were sealed and incubated at 30°C for at least 48 h. A change in the color of the bacteria to orange or brown signaled the production of HCN.

#### Cellulase production

To detect strains able to produce cellulase a carboxymethylcellulose (CMC) agar medium (Kasana et al., 2008) was used. Bacterial strains were prepared in liquid medium and incubated overnight, then 5 μL of each bacterial suspension were spot plated. The Petri dishes were incubated at 28°C for 48 h, then were flooded for 30–40 min with 1% hexadecyltrimethyl ammonium bromide. The appearance of an unstained area around the colony signaled cellulase production (Kasana et al., 2008).

#### Catalase production

A simple method suggested by Reiner (2010) was used. In brief, overnight grown bacterial strains were rinsed and resuspended in 1 mL 0.9% NaCl solution. Ten microliters of bacterial strains were spotted on a sterile and empty Petri dish, and a drop of 3% H_2_O_2_ solution was spotted on top of the bacterial strain. The strains were considered catalase positive if bubbles formed.

#### Exopolysaccharide production

To assess the ability of our bacterial strains to produce exopolysaccharides (EPS) the protocol proposed by Fusconi and Godinho (2002) was used. Bacteria liquid cultures (0.8 OD at 600 nm) were dot spotter on a mineral salt medium with 10% saccharose and incubated at 28°C for at least 48 h, the bacteria were considered EPS-positive if they are mucoid.

### Comparison of culture-dependent and culture-independent sequences

In a previous study (Mghazli et al., 2021), the bacterial diversity of phosphate wastes was characterized using Illumina MiSeq sequencing. The V4 region of the 16S rRNA gene sequences was targeted and revealed a huge number of sequences, totaling 8 million raw reads. In the present study, the TaxASS (Rohwer et al., 2018) workflow was used to BLAST the metabarcoding sequences against a customized database containing the sequences of the isolated strains. The Silva 132 database was chosen as a general reference database.

### Compatibility test for the selected strains

The bacterial strains used for the inoculation assay were subjected to antagonism tests to prevent any further inhibition effect in the *in planta* tests. Two methods were used, the first consisted of streaking two bacterial strains side-by-side on the same Petri dish containing YSA medium to test their diffusible compound effect (Jain et al., 2012). The second method consisted of spreading 100 μL of each bacterial strain on a Petri dish and place two dishes on top of each other to test their volatile compounds’ effect. The Petri plates were hermetically sealed with parafilm and incubated at 28°C for at least 3 days.

### Biocontrol—antifungal test

The antifungal activity through volatile compounds test was used as described by Ebadzadsahrai et al. (2020). In brief, bacterial strains were streaked on a YSA medium while a 6 mm diameter disc of the fungus (*Botrytis cinerea* and *Fusarium oxysporum*) was placed in the center of a Petri dish containing Potato Dextrose Agar (PDA) medium. The two plates were placed on top of each other and sealed, then incubated in the dark at 25°C for at least 7 days. The diffusible compounds antagonism test was also used as described by Hernández-León et al. (2015). To this end, bacterial strains were grown on solid LB medium for 24 h, before being filtered through a 0.22 μm filter, and 100 μL of the filtrate was spread on a Petri dish containing PDA medium. The plates were placed at 4°C for 2 h to help the diffusion of the bacterial filtrate through the medium, then a 6 mm fungal disc was placed in the center of each Petri dish and the plates were incubated in the dark at 25°C for 7 days. The percentage of inhibition of radial growth (PIRG) was calculated following the formula (Stracquadanio et al., 2020):


$$P\,I\,R\,G=\frac{R\,c-R\,s}{R\,c}\times 100$$

Where *Rc* is the radius of the fungal control and *Rs* is the radius of the fungal in presence of the bacterial strain.

### Metal tolerance tests

Tryptone Yeast (TY) medium (containing per liter: 5 g tryptone, 3 g yeast, and 0.8 g CaCl_2_) was supplemented with 200–900 mg/mL FeCl_2_.4H_2_O, 100–550 mg/L NaAsO_2_ [As (III)], 50–300 mg/L HgCl_2_, 10–300 mg/L CdCl_2_.4H_2_O, and 50–500 mg/L ZnCl_2_ each time. The plates were dot spotted with bacterial strains and incubated at 28°C for 10 days before final reading.

### Plant inoculation assay

*Lupinus albus* L. seeds were disinfected with 40% commercial bleach (12%) for 4 min and washed many times using sterile water. The seeds were then imbibed in sterile water for 4 h and germinated on a 0.6% water-agar medium. After 4 days, when the radicles start growing, the plantlets were placed in sieved non-sterile phosphate waste rock that served to isolate the bacterial strains.

Four treatments with five replicates were evaluated, three of which consisted of single inoculation, and one consisted of a consortium of the different strains. Non-inoculated plants were used as a control. Plants were grown in a greenhouse for 90 days. Fresh and dry weights and length of root and shoot parts were measured. Chlorophyll content was estimated following the protocol of Sharma et al. (2012). In brief, 0.5 g of fresh leaves was suspended in 25 mL of 80% acetone and 0.1% w/v CaCO_3_. The mixture was well-digested and mixed using the homogenizer, then centrifuged at 3000 rpm for 10 min at 4°C. The optical density of the supernatant was measured at 663, 645, and 470 nm. To determine the chlorophyll and the carotenoid contents, the following formula was used (Lichtenthaler and Wellburn, 1983):


$$\text{Chl a}=11.75{\text{ OD}}_{663}-2.350{\text{ DO}}_{645}$$

$$\text{Chl b}=18.61{\text{ OD}}_{645}-3.96{\text{ DO}}_{663}$$

$$\text{Car}=(1000{\text{ OD}}_{470}-2.270\text{ Chl a}-81.4\text{ Chl b)}/227$$

Where Chl a is the chlorophyll a content, Chl b is the chlorophyll b content, Car is the carotenoid content.

All tests were repeated three times and data was analyzed using R (V 4.3) using variance analysis (ANOVA) and Tukey test (*p* < 0.05). Graphs visualization was done using ggplot2 package.

## Results

### Isolation and molecular characterization of the isolated bacterial community

A total of 41 bacterial colonies were isolated from the phosphate mine wastes. Their sequences were BLASTed within NCBI database, and similar type strain sequences (with different similarity percentages) were then aligned with our strains using Mega X (Figure 1). *Actinobacteria* was the dominant phylum with 53.7% bacterial strains (22 out of 41), while 26.8% of the bacterial strains (11 out of 41) were affiliated to *Firmicutes* phylum, and only 19.5% (8 out of 41) was affiliated to *Proteobacteria* phylum. The most abundant genus was *Microbacterium* with 10 representatives, followed by *Bacillus* with 8 representatives. The remaining strains were affiliated to *Acinetobacter*, *Agrococcus*, *Brevibacterium*, *Neobacillus*, *Paenibacillus*, *Peribacilllus*, *Pseudarthrobacter*, *Stenotrophomonas*, and *Raoultella* genera, with 2, 6, 1, 1, 1, 1, 5, 5, and 1 representatives, respectively (Supplementary Table 2). The comparison between culture-dependent and culture-independent bacteria using TaxASS software revealed that all isolated bacteria were already present in the metabarcoding sequence data (Supplementary Figure 1).

### Growth in response to abiotic stresses

**FIGURE 2:**
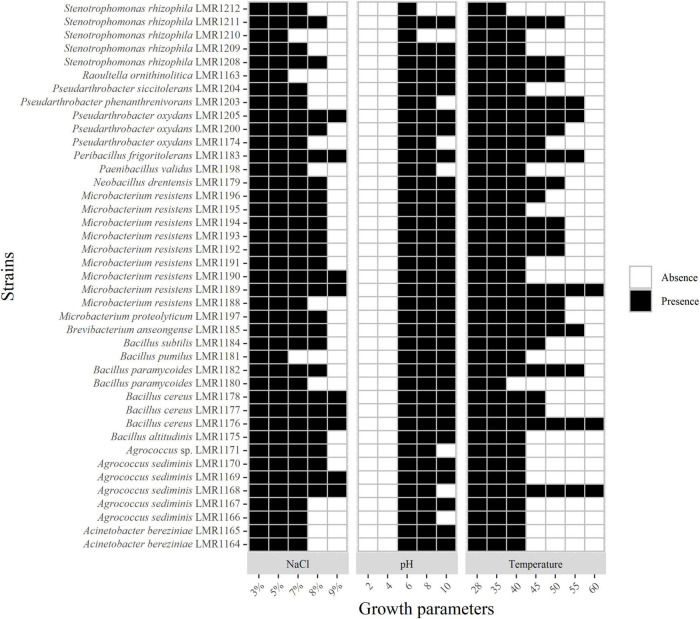
Heatmap of the capacity of the 41 isolated strains to tolerate different concentrations of NaCl and different pH and temperature. Presence and absence indicate the presence or the absence of growth.

All isolates were able to tolerate up to 5% of NaCl; 38 strains were able to tolerate 7% of NaCl; 26 strains tolerated 8% of NaCl while only 9 strains were able to grow in the presence of 9% of NaCl but none of the isolated strains was able to grow above this concentration (Figure 2).

As mentioned in Supplementary Table 1, the pH of phosphate mining wastes in this study is alkaline (8.1). None of the 41 bacterial strains was able to grow at a pH below 4; the optimal pH of all strains was 7. All representatives of *Acinetobacter*, *Bacillus*, *Microbacterium* and the strains similar to *Brevibacterium*, *Peribacillus*, and *Raoultella* genera were able to grow at pH 10, whereas none of the isolated bacteria was able to grow above this pH (Figure 2).

All strains were able to tolerate temperatures up to 40°C except *Stenotrophomonas rhizophila* LMR1212 and *Bacillus paramycoides* LMR1180 (Figure 2). Only three strains were able to grow at 60°C: *Agrococcus sediminis* LMR1168, *Bacillus cereus* LMR1176, and *Microbacterium resistens* LMR1189.

### Screening for plant-growth promoting activities

**FIGURE 3:**
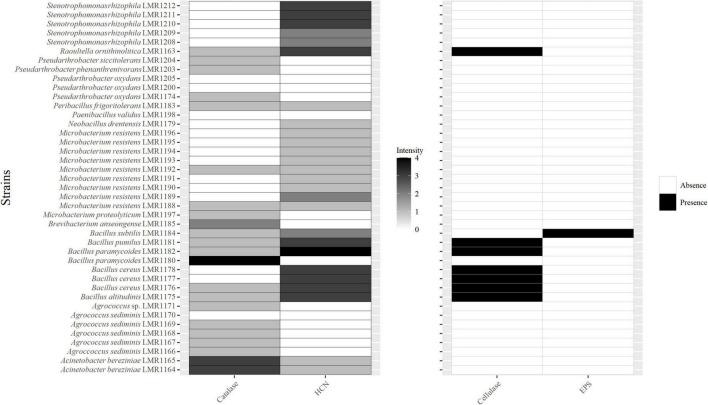
Heatmap of the capacity of the 41 isolated bacterial strains to produce catalase; hydrogen cyanide (HCN); cellulase and exopolysaccharides. For catalase and HCN; different intensities are presented, 0 for no activity, 1 for low activity, 2 for medium activity, 3 for high activity, and 4 for very high activity.

Twenty-two isolates were positive for catalase with different intensities (Figure 3). *Bacillus paramycoides* LMR1180 showed the best catalase activity, followed by the two strains of *Acinetobacter bereziniae* (LMR1164 and LMR1165), and by *Brevibacterium anseongense* LMR1185, whereas the remaining 18 strains showed quite low catalase activity.

Twenty-six strains were positive for HCN (Figure 3). The highest activity was observed with *Bacillus paramycoides* LMR1182, followed by five strains of *Bacillus* (*Bacillus altitudinis* LMR1175, *Bacillus cereus* LMR1176; LMR1177 and LMR1178, and *Bacillus pumilus* LMR1181), *Raoultella ornithinolytica* LMR1163, and 3 strains of *Stenotrophomonas rhizophila* (LMR1210; LMR1211; and LMR1212). *Bacillus subtilis* LMR1184 and *Microbacterium resistens* LMR1189 showed medium production, while the remaining 14 strains showed poor HCN production.

Only strains of *Raoultella* (*Raoultella ornithinolytica* LMR1163) and *Bacillus* (*Bacillus paramycoides* LMR1182; *Bacillus pumilus* LMR1181; *Bacillus cereus* LMR1176-1178 and *Bacillus altitudinis* LMR1175) showed a positive reaction to the cellulase activity (Figure 3), while only *Bacillus subtilis* LMR1184 was EPS positive.

**FIGURE 4:**
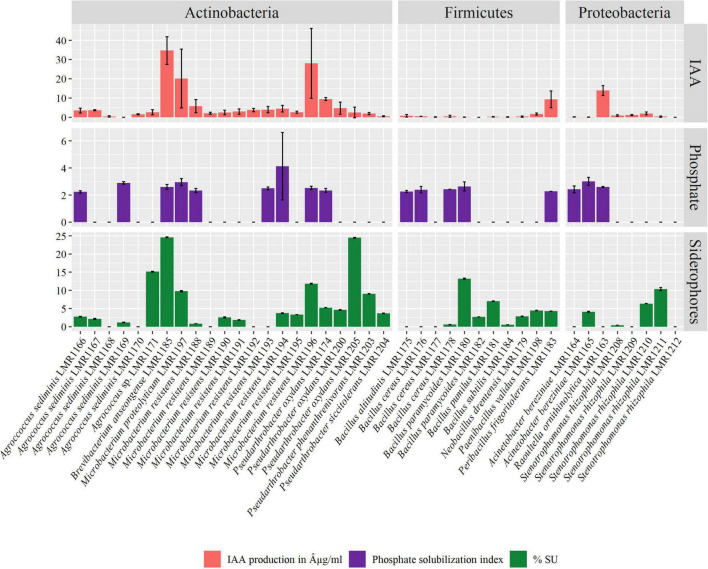
Plant growth promoting activities of the 41 isolated strains. Indole-3-acetic acid (IAA) production in μg/mL, phosphate solubilization index, and siderophore production in percent siderophore unit (%SU).

The majority of our bacterial strains tested positive for IAA production (Figure 4). IAA production ranged from 0.005 to 34.68 μg/mL. Among representatives of *Actinobacteria* phylum, all strains except *Agrococcus sediminis* LMR1169 were able to produce different quantities of IAA, while among *Firmicutes* representatives, *Peribacillus frigoritolerans* LMR1183 produced 9.3 μg/mL of IAA, and as for *Proteobacteria* phylum, *Raoultella ornithinolytica* LMR1163 produced the highest concentration of IAA, i.e., exceeding 13 μg/mL. The highest IAA production was recorded in *Brevibacterium anseongense* LMR1185 (34.68 μg/mL), followed by *Microbacterium resistens* LMR1196 (28.05 μg/mL) and *Microbacterium proteolyticum* LMR1197 (11.76 μg/mL).

Seventeen strains were able to solubilize phosphate with a solubilization index ranging from 2.24 for *Agrococcus sediminis* LMR1166 to 4.13 for *Microbacterium resistens* LMR1194 (Figure 4). Nine strains of *Actinobacteria* phylum, five of *Firmicutes* phylum, and three of *Proteobacteria* phylum were able to solubilize different amounts of TCP.

Finally, 29 strains were able to produce siderophores, with production ranging from 0.38 to 24.6% SU (Figure 4). *Brevibacterium anseongense* LMR1185 produced the largest quantity of siderophores, followed by *Pseudarthrobacter oxydans* LMR1205 (24.5% SU). Four other strains (*Agrococcus* sp. LMR1171, *Bacillus paramycoides* LMR1180, *Microbacterium resistens* LMR1196, and *Stenotrophomonas rhizophila* LMR1211) produced more than 10% SU.

### Selection of strains for in-planta assay and further *in vitro* analyses

Based on all above results, three phylogenetically diversified strains, *Bacillus paramycoides* LMR1180, *Brevibacterium anseongense* LMR1185, and *Stenotrophomonas rhizophila* LMR1211, were chosen for the plant inoculation assay. The selection was based on all tests with a focus on PGP activities and discarding all strains with a potential pathogenic activity on human. These chosen bacteria were tested for other activities (antifungal, metal tolerance, and compatibility tests; Supplementary Table 3). The three selected bacteria were tested for their antagonism effects to eliminate any potential antagonism between strains that may affect the growth of the plant. No growth inhibition effect was detected between strains, neither *via* diffusible compounds nor through volatile compounds.

All the selected strains were able to inhibit the growth of the two fungi (*Fusarium oxysporum* and *Botrytis cinerea*) through volatile and diffusible compounds. *Bacillus paramycoides* LMR1180 showed the best effect against both fungi *via* diffusible compounds, but presents also quite good effects *via* volatile compounds. *Brevibacterium anseongense* LMR1185 and *Stenotrophomonas rhizophila* LMR1211 had the best effects against *Fusarium oxysporum* and *Botrytis cinerea*, respectively, *via* volatile compounds (Table 1).

**TABLE 1:** Effects of the three selected strains (*Bacillus paramycoides* LMR1180, *Brevibacterium anseongense* LMR1185, and *Stenotrophomonas rhizophila* LMR1211) on the radial growth of two fungi (*Botrytis cinerea* and *Fusarium oxysporum*).

	Diffusible compounds		Volatile compounds	
		
	*Fusarium oxysporum*	*Botrytis cinerea*	*Fusarium oxysporum*	*Botrytis cinerea*
*Bacillus paramycoides* LMR1180	7.69 ± 0.3%	14.44 ± 0.1%	10.78 ± 0.25%	51.96 ± 0.55%
*Brevibacterium anseongense* LMR1185	5.77 ± 0.35%	10 ± 0.15%	12.74 ± 0.65%	42.16 ± 0.05%
*Stenotrophomonas rhizophila* LMR1211	3.85 ± 0.1%	4.44 ± 0.1%	7.84 ± 0%	53.92 ± 0.35%

The inhibition was evaluated *via* diffusible and volatile compounds, and is represented in percentage.

The three selected strains showed a quite high metal tolerance to all tested metals (NaAsO_2_, FeCl_2_, and HgCl_2_) except for CdCl_2_ where *Bacillus paramycoides* LMR1180 and *Brevibacterium anseongense* LMR1185 could not tolerate more than 20 and 10 mg/L, respectively, or for ZnCl_2_ where *Bacillus paramycoides* LMR1180 could not tolerate more than 50 mg/L (Table 2).

**TABLE 2:** Table representing the highest concentrations of toxic metals tolerated by the three selected strains (*Bacillus paramycoides* LMR1180, *Brevibacterium anseongense* LMR1185, and *Stenotrophomonas rhizophila* LMR1211).

	*Bacillus paramycoides* LMR1180	*Brevibacterium anseongense* LMR1185	*Stenotrophomonas rhizophila* LMR1211
NaAsO_2_ (As (III))	550 mg/L	550 mg/L	550 mg/L
CdCl_2_ (Cd)	20 mg/L	10 mg/L	170 mg/L
FeCl_2_ (Fe)	600 mg/L	600 mg/L	800 mg/L
HgCl_2_ (Hg)	100 mg/L	100 mg/L	200 mg/L
ZnCl_2_ (Zn)	50 mg/L	200 mg/L	400 mg/L

### Plant inoculation assay

Non-sterilized phosphate waste rocks were used for the inoculation test. Five germinated plantlets were used per test, and along the inoculation assay, the plantlets were sprinkled with distilled water every other day. After 90 days, different plant parameters were measured.

**FIGURE 5:**
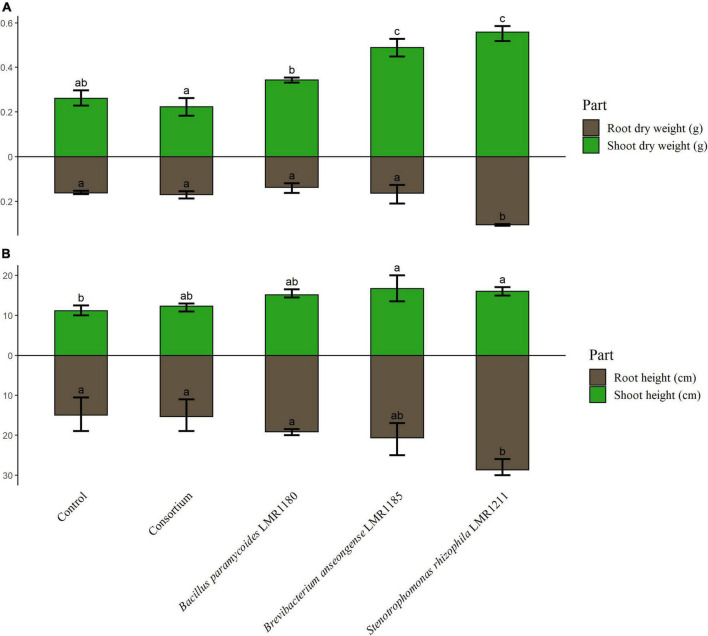
The measurement of control and inoculated *Lupinus albus* plants using the three selected strains (*Bacillus paramycoides* LMR1180, *Brevibacterium anseongense* LMR1185, and *Stenotrophomonas rhizophila* LMR1211). The dry weight of shoot and root parts **(A)** is represented in g, and the height of shoot and root parts **(B)** in cm after 90 days under greenhouse conditions. The letters (a, b, and c) present the compact display letters (CDL). Values in each bar followed by the same letter are not significantly different at 0.05% (Tukey’s).

The shoot weights of plants treated with *Brevibacterium anseongense* LMR1185, and *Stenotrophomonas rhizophila* LMR1211 were similar and significantly higher than non-inoculated plants (Figure 5). No significant effects were observed for plants treated with *Bacillus paramycoides* LMR1180 or with the consortium compared to the control. Only plants treated with *Stenotrophomonas rhizophila* LMR1211, showed also significant differences in the root dry weight compared to the non-inoculated plants. Similarly, plants treated with *Stenotrophomonas rhizophila* LMR1211 and *Brevibacterium anseongense* LMR1185 had a significantly higher shoots than the control (Figure 5).

**FIGURE 6:**
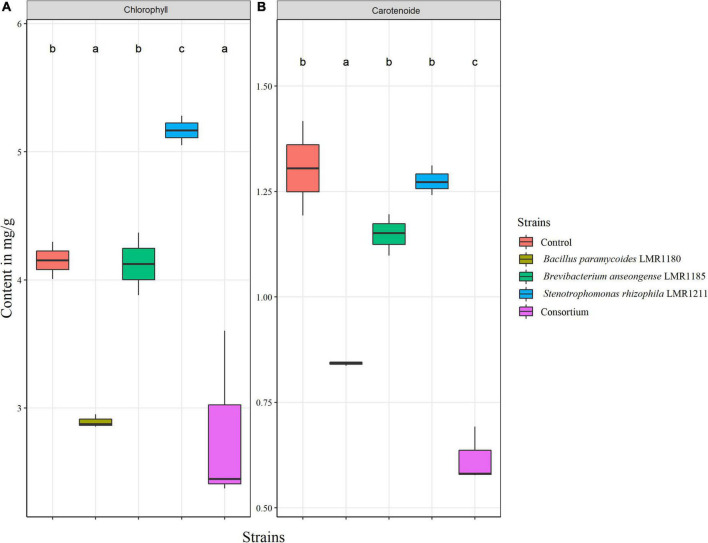
The estimation of total chlorophyll **(A)** and carotenoid **(B)** content in control and inoculated *Lupinus albus* plants using the three selected strains (*Bacillus paramycoides* LMR1180, *Brevibacterium anseongense* LMR1185, and *Stenotrophomonas rhizophila* LMR1211) in mg/g of biomass after 90 days under greenhouse conditions. The letters (a, b, and c) present the compact display letters (CDL). Values in each bar followed by the same letter are not significantly different at 0.05% (Tukey’s).

The total chlorophyll content was only significantly higher for plants treated with *Stenotrophomonas rhizophila* LMR1211 (5.16 ± 0.12 mg/g) compared to the control, while no significant difference was observed in the carotenoid content from *Stenotrophomonas rhizophila* LMR1211 and *Brevibacterium anseongense* LM1185 compared to the control (Figure 6). We also note that *Bacillus paramycoides* LMR1180 and the consortium showed the lowest chlorophyll and carotenoid contents.

## Discussion

In Morocco, the development of vegetation in the harsh conditions of phosphate mine wastes in a semi-arid climate (requiring drought-, heat-, metal-, and salt-tolerant plants adapted to high-velocity winds) is key to the bioremediation by revegetation of these wastes dumps. As bacterial communities play a key role in the development of soil and plants, understanding bacterial structure, diversity, and their potential plant growth activities is the first step toward revegetation of phosphate tailings (Nannipieri et al., 2003; Luo et al., 2018). The objectives were to isolate and characterize the potential indigenous PGPB from phosphate wastes and select the most efficient strains for the growth of plants. To this end, forty-one bacterial strains were isolated, identified, and screened for their PGP activities *in vitro*. The best performing bacteria were also tested *in planta*. A very limited number of studies have focused on isolating the bacterial community from phosphate wastes and they mainly studied the phosphate solubilizing activity in bacteria for their use as phosphate biofertilizers (Hamdali et al., 2008, 2012; Yang et al., 2012; Aliyat et al., 2020; Benbrik et al., 2020; Qarni et al., 2021). To our knowledge, this is one of the first studies on the effect of indigenous bacteria on plant growth for revegetation purposes of phosphate wastes.

Among the 11 bacterial genera isolated in this study, only 4 were retrieved in precedent works in phosphate mine wastes: *Acinetobacter*, *Bacillus*, *Brevibacterium*, and *Raoultella* (Yang et al., 2012; Benbrik et al., 2020; Qarni et al., 2021). However, other genera such as *Micromonospora*, *Streptomyces*, *Pseudomonas*, *Sphingobacterium*, *Staphylococcus*, and *Serratia* were not identified in the present study (Hamdali et al., 2008, 2012; Yang et al., 2012; Benbrik et al., 2020; Qarni et al., 2021). This could be due to the isolation medium used or to the type of phosphate, and process implicated in generating these wastes.

The majority of the isolated strains were able to tolerate high temperatures, substrate salinity and alkalinity. This can be due to the presence of arid to semi-arid climate of study site, which results in a high evaporation rate causing serious drought stress and salinity in the surface soil.

Among the isolated strains, six *Bacillus* strains and one *Raoultella* were able to produce cellulase, an enzyme that could help in the bioremediation of de-structured soils by increasing seeds germination and/or by playing a key role in roots development (Kuhad et al., 2011). While only *Bacillus subtilis* LMR1184 was able to produce EPS. This is in accordance with the results of Moghannem et al. (2018) showing that different species of *Bacillus* genus were able to produce EPS. These metabolites, produced by bacteria, could help the development of plants in harsh condition by segregating metals, alleviating salinity, improving humidity of soil and soil aggregation (Sandhya and Ali, 2015).

Many of the isolated strains were able to produce HCN and catalase. *Bacillus paramycoides* LMR1182 produced the most HCN quantity, while *Bacillus paramycoides* LMR1180 exhibited the highest catalase production. In general, *Bacillus* spp. are well-known for their HCN (Walia et al., 2014; Brzezinska et al., 2020) and catalase production (Fibriarti et al., 2021). HCN is known for its capacity to counter the development and growth of plant pathogens (Nandi et al., 2017). Besides their biocontrol capacity, HCN-producing bacteria can enhance plant development by increasing the quantity of bioavailable phosphate in soil (Rijavec and Lapanje, 2016). Whereas catalase-producing bacteria can protect themselves and the surrounding soil, including plants from hydrogen peroxide and reactive oxygen species, responsible for cell wall degradation when present in excess (Reiner, 2010). Stress suppressing catalase can break down H_2_O_2_ molecules, thereby providing more oxygen to the soil (Singh and Kumar, 2019).

More than 95% of the isolated strains (39 strains) were able to produce IAA, which enhances plant growth by improving seed germination, shoot elongation, and the formation of root hairs which increases water and nutrient uptake (Small and Degenhardt, 2018; Ullah and Bano, 2019). In the present work, IAA production ranged from 0.005 μg/mL for *Bacillus paramycoides* LMR1180 to 34.7 μg/mL for *Brevibacterium anseongense* LMR1185 with 12 strains exceeding 10 μg/mL. This is in accordance with findings reported by Aliyat et al. (2020) where the production of IAA by strains isolated from a Moroccan phosphate sludge ranged from 1.37 to 43.8 μg/mL, with 5 strains out of 27 exceeding 10 μg/mL.

Alkaline soils are more subject to iron deficiency, hence affecting the growth of plants in these soils (Balparda et al., 2021). Iron solubility in soil is proportional to soil pH, the higher the pH is, the less soluble iron is (Mann et al., 2017; Singh et al., 2022). Siderophore producing bacteria alleviate iron deficiency in plants and minimize negative effects of saline soils such as chlorosis, as well as increase chlorophyll content (Ferreira et al., 2019; Silambarasan et al., 2020; Singh et al., 2022). In the present study, 29 strains were able to produce siderophores. *Bacillus paramycoides* LMR1180, *Brevibacterium anseongense* LMR1185, and *Stenotrophomonas rhizophila* LMR1211, produced the most siderophores in their respective phyla with 13.2, 24.7, and 10.8% SU, respectively. This is in agreement with research by El-Sersawy et al. (2021) and Liu et al. (2022) showing, respectively, that *Bacillus paramycoides* and *Stenotrophomonas rhizophila* can produce siderophores.

As phosphorus predominates in phosphate mine wastes, our bacteria do not generally display the need to solubilize additional phosphate for their cell machinery. As described by Michas et al. (2021), the phosphate solubilizing bacteria community changes in parallel with variations in soil composition, especially the carbon to phosphorus (C:P) ratio. These factors could explain the small number of bacterial isolates able to solubilize tricalcium phosphate in the present study.

The preliminary testing revealed that the majority of the isolated strains displayed good PGP activities. This is also the first time the recently described species *Brevibacterium anseongense* (Jung et al., 2018) and *Agrococcus sediminis* (Qu et al., 2020) were studied for their PGP activities.

To go further in this study, three phylogenetically diversified strains with excellent performance in *in vitro* analyses were subjected to metal resistance, biocontrol activities and a further pot experiment. The three selected strains, LMR1180, LMR1185, and LMR1211, showed high similarity to *Bacillus paramycoides* MCCC 1A04098, *Brevibacterium anseongense* Gsoil 188, and *Stenotrophomonas rhizophila* e-p10, respectively (Supplementary Table 2). The present works’ additional *in planta* test findings aided in determining how well the chosen strains can promote plant growth in stressed environments, contaminated with excess concentration of phosphate associated with nutrient-poor soils.

As phosphate mining wastes in Morocco may contain toxic metallic elements (Hakkou et al., 2009; Ouakibi et al., 2013), the resistance of the strains chosen for pot experiments were tested for some metals and metalloids. All these strains performed similarly in As(III), with a tolerance reaching 550 mg/L of NaAsO_2_. For Cd, *Stenotrophomonas rhizophila* LMR1211 was the most tolerant strain (until 170 mg/L of CdCl_2_), exceeding the concentration of Gao et al. (2020). *Stenotrophomonas rhizophila* LMR1211 showed also the most tolerance capacity for Fe, Hg, and Zn with concentrations reaching 800, 200, and 400 mg/L, respectively. This is in agreement with results of Ryan et al. (2009) describing *Stenotrophomonas rhizophila* as a potent multi-metal resistant strain.

Another mechanism that aids in plant establishment under stressed conditions, is the capacity of some bacteria to act as biocontrol agents, which could limit the negative effects of numerous pathogenic agents (Glick, 2012). Indeed, Mahmud et al. (2021) demonstrated the positive effect that biocontrol agents play in plant-growth promotion through their antagonistic relationship with pathogenic microorganisms in general, and fungi specifically. In the present study, *Bacillus paramycoides* LMR1180 performed the best in mycelial growth inhibition *via* diffusible compounds, and was able to inhibit the growth of *Fusarium oxysporum* and *Botrytis cinerea* by 7.7 and 14.4%, respectively. A similar study has shown that *Bacillus paramycoides* was able to limit *F. oxysporum* growth by more than 52% (El-Sersawy et al., 2021). As for the volatile compounds, *Stenotrophomonas rhizophila* LMR1211 inhibited *Botrytis cinerea* by 53.9%. Ryan et al. (2009) reported that *Stenotrophomonas rhizophila* inhibited also more than 9% of *Rhizoctonia solani* mycelium growth. *Brevibacterium anseongense* LMR1185 limited the growth of *F. oxysporum* by 12.7%. This is, to our knowledge, the first time *Brevibacterium anseongense* was tested for its biocontrol activity.

The in-pot experiments were performed with the white lupin (*Lupinus albus* L.), a Mediterranean annual nitrogen fixing legume (Huyghe, 1997). This plant, was shown to be hyperaccumulator in contaminated sites (González et al., 2021; Quiñones et al., 2021) making it a good candidate for bioremediation schemes and the establishment of a vegetative cover on top of phosphate wastes. In the current work, *Stenotrophomonas rhizophila* LMR1211 enhanced significantly the shoot and root dry weight as well as the root height. This is in line with the reported results by Ryan et al. (2009) suggesting that *Stenotrophomonas rhizophila* entertains a beneficial microorganism-plant interaction. Additionally, the inoculation with *Brevibacterium anseongense* LMR1185 showed a significant increase in shoot dry weight and height, and in root height. This is in accordance with the findings of González et al. (2021) showing that the inoculation of *Lupinus albus* L. with a species of *Brevibacterium* (*Brevibacterium frigoritolerans*) had a significant effect on shoots and roots parts parameters. Whilst neither the inoculation with *Bacillus paramycoides* LMR1180 nor with the consortium showed any improvement on *Lupinus albus* L. dry weight or height over the non-inoculated control. The use of a consortium, suggested by different previous study, was conducted in the hope to cumulate the PGP effects of each bacterium (Adams et al., 2014; Zhang et al., 2020). However, this assumption was not correct in the present study.

Biologists recognized the relevance of measuring the chlorophyll content from plant leaves. It has been shown that the content of leaves chlorophyll is correlated with the plant photosynthetic capacity (Buttery and Buzzell, 1977) thus helping plant growth and development (Rojas-Tapias et al., 2012). *Stenotrophomonas rhizophila* LMR1211 increased significantly the chlorophyll content in *Lupinus albus* leaves, which may explain the great development observed in this study, especially dry weight and length. The results observed in the consortium could be explained by the PGP activities antagonism. As a hypothesis, we wanted to check if the PGP activities of the three strains were kept when in consortium, and it has been shown that both *Stenotrophomonas rhizophila* LMR1211 and *Brevibacterium anseongense* LMR1185 inhibited the production of IAA by *Bacillus paramycoides* LMR1180, which may explain the poor development of the consortium root system, impacting the shoots development especially and the plant growth in general. While none of the two other activities (siderophore production or phosphate solubilization) were significantly affected in the consortium.

## Conclusion

To help the establishment of a vegetative cover on phosphate mining wastes, this study explored the plant growth activity of indigenous bacteria. Forty-one bacterial strains were isolated and identified and the three best-performing strains (*Bacillus paramycoides* LMR1180, *Brevibacterium anseongense* LMR1185, and *Stenotrophomonas rhizophila* LMR1211) were chosen for further *in vitro* and *in planta* analyses. *Stenotrophomonas rhizophila* LMR1211 exhibited the best performance in promoting white lupin growth, a Mediterranean annual legume, followed by *Brevibacterium anseongense* LMR1185. These two strains should be put forward for further analyses to prove their positive impact on plant growth under stressed conditions and ultimately be used as a potential biofertilizer. To our knowledge, this is the first time *Brevibacterium anseongense* was reported to have good PGP activities and the ability to promote plant growth in pot experiments. The use of these strains as biofertilizers could represent an economical and eco-friendly method for the rehabilitation of phosphate mining waste in Morocco.

## Data availability statement

The datasets presented in this study can be found in online repositories. The names of the repository/repositories and accession number(s) can be found below: https://www.ncbi.nlm.nih.gov/, MZ853464.2; MZ853485.2; MZ853448.2; MZ853453.2; MZ853480.2; MZ853474.2; MZ853458.2; MZ853482.1; MZ853455.2; MZ853469.2; MZ853459.2; MZ853445.2; MZ853444.2; MZ853449.2; MZ853468.2; MZ853483.2; MZ853471.2; MZ853465.2; MZ853466.2; MZ853478.2; MZ853456.2; MZ853462.2; MZ853470.2; MZ853475.2; MZ853439.2; MZ853442.2; MZ853476.2; MZ853479.1; MZ853450.2; MZ853481.2; MZ853451.2; MZ853447.2; MZ853486.2; MZ853452.2; MZ853457.2; MZ853484.2; MZ853443.1; MZ853463.1; MZ853477.1; MZ853440.1; MZ853438.1.

## Author contributions

NM, OB, RH, and LS designed and contributed to the conception of the work. NM performed the sampling along with the experiments and wrote the first draft of the manuscript. RZ contributed to the experiments. NM and LS analyzed the data. OB contributed to the data interpretation. All authors reviewed and approved the final version of this manuscript.
