# Supplementary material for: Characterization of plant growth promoting activities of indigenous bacteria of phosphate mine wastes, a first step toward revegetation
Source: Front Microbiol. 2022 Dec 15;13:1026991. doi: 10.3389/fmicb.2022.1026991 (PMC9798287; doi:10.3389/fmicb.2022.1026991)
Supplement: Supplementary file 1 [file Data_Sheet_1.docx]

Supplementary Material

*Table S1: Physico-chemical parameters of the phosphate waste rock from Mghazli et al. (2021)*

|  | Phosphate waste rock | unit |
| --- | --- | --- |
| pH | 8.1 ± (0) | - |
| TOC* | 0.32 ± (0) | % |
| N | 0.02 ± (0) | % |
| SiO_2_ | 117800 ± (6138.95) | mg/Kg |
| P_2_O_5_ | 79333.33 ± (2072.57) | mg/Kg |
| Fe_2_O_3_ | 44366.67 ± (659.97) | mg/Kg |
| CaO | 395933.33 ± (4805.78) | mg/Kg |
| MgO | 25000 ± (4329.74) | mg/Kg |
| K_2_O | 5200 ± (1363.82) | mg/Kg |
| TiO_2_ | 1300 ± (326.6) | mg/Kg |
| Al_2_O_3_ | 20966.67 ± (1975.4) | mg/Kg |

*TOC: Total Organic Carbon

***Table S2****: Phylogenetically similar reference sequences and their identity percentage with the isolated strains*

| Strain | Sequence reference | Query Length | Similarity | Accession number |
| --- | --- | --- | --- | --- |
| *Acinetobacter bereziniae* strain LMR1164 | *Acinetobacter bereziniae* strain ATCC 17924 (NR_117625.1) | 1401 | 100% | MZ853464.2 |
| *Acinetobacter bereziniae* strain LMR1165 | *Acinetobacter bereziniae* strain ATCC 17924 (NR_117625.1) | 1267 | 100% | MZ853485.2 |
| *Agrococcus sediminis* strain LMR1166 | *Agrococcus sediminis* strain NS18 (NR_174209.1) | 1389 | 99.50% | MZ853448.2 |
| *Agrococcus sediminis* strain LMR1167 | *Agrococcus sediminis* strain NS18 (NR_174209.1) | 1347 | 99.55% | MZ853453.2 |
| *Agrococcus sediminis* strain LMR1168 | *Agrococcus sediminis* strain NS18 (NR_174209.1) | 1389 | 99.50% | MZ853480.2 |
| *Agrococcus sediminis* strain LMR1169 | *Agrococcus sediminis* strain NS18 (NR_174209.1) | 1373 | 99.41% | MZ853474.2 |
| *Agrococcus sediminis* strain LMR1170 | *Agrococcus sediminis* strain NS18 (NR_174209.1) | 1388 | 99.57% | MZ853458.2 |
| *Agrococcus* sp. LMR1171 | *Agrococcus* *sediminis* strain NS18 (NR_174209.1) | 1212 | 97.73% | MZ853482.1 |
| *Bacillus altitudinis* strain LMR1175 | *Bacillus altitudinis* strain 41KF2b (NR_042337.1) | 1424 | 100% | MZ853455.2 |
| *Bacillus cereus* strain LMR1176 | *Bacillus cereus* strain ATCC 14579 (NR_074540.1) | 1425 | 99.86% | MZ853469.2 |
| *Bacillus cereus* strain LMR1177 | *Bacillus cereus* strain ATCC 14579 (NR_074540.1) | 1423 | 100% | MZ853459.2 |
| *Bacillus cereus* strain LMR1178 | *Bacillus cereus* strain ATCC 14579 (NR_074540.1) | 1412 | 99.93% | MZ853445.2 |
| *Bacillus paramycoides* strain LMR1180 | *Bacillus paramycoides* strain MCCC 1A04098 (NR_157734.1) | 1413 | 99.93% | MZ853444.2 |
| *Bacillus pumilus* strain LMR1181 | *Bacillus pumilus* strain ATCC 7061 (NR_043242.1) | 1445 | 100% | MZ853449.2 |
| *Bacillus* *paramycoides* strain LMR1182 | *Bacillus paramycoides* strain MCCC 1A04098 (NR_157734.1) | 1423 | 100% | MZ853468.2 |
| *Peribacillus frigoritolerans* strain LMR1183 | *Peribacillus frigoritolerans* strain DSM 8801 (NR_117474.1) | 1136 | 99.65% | MZ853483.2 |
| *Bacillus* *subtilis* strain LMR1184 | *Bacillus subtilis subsp. subtilis* strain 168 ( NR_102783.2) | 1458 | 99.79% | MZ853471.2 |
| *Brevibacterium anseongense* strain LMR1185 | *Brevibacterium anseongense* strain Gsoil 188 (NR_165697.1) | 1393 | 99.64% | MZ853465.2 |
| *Microbacterium resistens* strain LMR1188 | *Microbacterium resistens* strain DMMZ 1710 (NR_026437.1) | 1393 | 99.57% | MZ853466.2 |
| *Microbacterium resistens* strain LMR1189 | *Microbacterium resistens* strain DMMZ 1710 (NR_026437.1) | 1358 | 99.56% | MZ853478.2 |
| *Microbacterium resistens* strain LMR1190 | *Microbacterium resistens* strain DMMZ 1710 (NR_026437.1) | 1361 | 99.48% | MZ853456.2 |
| *Microbacterium resistens* strain LMR1191 | *Microbacterium resistens* strain DMMZ 1710 (NR_026437.1) | 1392 | 99.50% | MZ853462.2 |
| *Microbacterium resistens* strain LMR1192 | *Microbacterium resistens* strain DMMZ 1710 (NR_026437.1) | 1382 | 99.49% | MZ853470.2 |
| *Microbacterium resistens* strain LMR1193 | *Microbacterium resistens* strain DMMZ 1710 (NR_026437.1) | 1349 | 99.63% | MZ853475.2 |
| *Microbacterium resistens* strain LMR1194 | *Microbacterium resistens* strain DMMZ 1710 (NR_026437.1) | 1386 | 99.57% | MZ853439.2 |
| *Microbacterium resistens* strain LMR1195 | *Microbacterium resistens* strain DMMZ 1710 (NR_026437.1) | 1369 | 99.63% | MZ853442.2 |
| *Microbacterium resistens* strain LMR1196 | *Microbacterium resistens* strain DMMZ 1710 (NR_026437.1) | 1356 | 99.63% | MZ853476.2 |
| *Microbacterium* *proteolyticum* strain LMR1197 | *Microbacterium proteolyticum* strain RZ36 (NR_135869.1) | 1293 | 99.07% | MZ853479.1 |
| *Neobacillus drentensis* strain LMR1179 | *Neobacillus drentensis* strain NBRC 102427 (NR_114085.1) | 1430 | 99.78% | MZ853450.2 |
| *Paenibacillus validus* strain LMR1198 | *Paenibacillus validus* strain JCM 9077 (NR_040892.1) | 1059 | 99.53% | MZ853481.2 |
| *Pseudarthrobacter* *oxydans* strain LMR1174 | *Pseudarthrobacter oxydans* strain DSM 20119 (NR_026236.1) | 1342 | 99.63% | MZ853451.2 |
| *Pseudarthrobacter oxydans* strain LMR1200 | *Pseudarthrobacter oxydans* strain DSM 20119 (NR_026236.1) | 1361 | 99.63% | MZ853447.2 |
| *Pseudarthrobacter phenanthrenivorans* strain LMR1203 | *Pseudarthrobacter phenanthrenivorans* Sphe3 (NR_074770.2) | 1097 | 99.82% | MZ853486.2 |
| *Pseudarthrobacter* *siccitolerans* strain LMR1204 | *Pseudarthrobacter siccitolerans* strain 4J27 (NR_108849.1) | 848 | 99.65% | MZ853452.2 |
| *Pseudarthrobacter* *oxydans* strain LMR1205 | *Pseudarthrobacter oxydans* strain DSM 20119 (NR_026236.1) | 1398 | 99.29% | MZ853457.2 |
| *Raoultella* *ornithinolytica* strain LMR1163 | *Raoultella ornithinolytica* strain ATCC 31898 (NR_114502.1) | 1242 | 99.84% | MZ853484.2 |
| *Stenotrophomonas rhizophila* strain LMR1208 | *Stenotrophomonas rhizophila* strain e-p10 (NR_121739.1) | 1417 | 98.87% | MZ853443.1 |
| *Stenotrophomonas rhizophila* strain LMR1209 | *Stenotrophomonas rhizophila* strain e-p10 (NR_121739.1) | 1405 | 99.63% | MZ853463.1 |
| *Stenotrophomonas* *rhizophila* strain LMR1210 | *Stenotrophomonas rhizophila* strain e-p10 (NR_121739.1) | 1418 | 99.36% | MZ853477.1 |
| *Stenotrophomonas rhizophila* strain LMR1211 | *Stenotrophomonas rhizophila* strain e-p10 (NR_121739.1) | 1418 | 99.36% | MZ853440.1 |
| *Stenotrophomonas rhizophila* strain LMR1212 | *Stenotrophomonas rhizophila* strain e-p10 (NR_121739.1) | 1411 | 99.50% | MZ853438.1 |

*Table S3: Table summarizing the different activities/criteria considered in the choice of the three selected strains (highlighted)*

| *Strains* | *NaCl tolerance* | *pH tolerance* | *T° tolerance* | *IAA production* | *Siderophore production* | *HCN production* | *Catalase production* | *Cellulase production* | *Risk group*  *(based on LPSN)** |
| --- | --- | --- | --- | --- | --- | --- | --- | --- | --- |
| *Acinetobacter bereziniae* strain LMR1164 | *+* | *++* | *-* | *-* | *-* | *-* | *++* | *-* | *2* |
| *Acinetobacter bereziniae* strain LMR1165 | *+* | *++* | *-* | *-* | *+* | *-* | *++* | *-* | *2* |
| *Agrococcus sediminis* strain LMR1166 | *+* | *+* | *-* | *-* | *+* | *-* | *-* | *-* | **** |
| *Agrococcus sediminis* strain LMR1167 | *+* | *++* | *-* | *-* | *+* | *-* | *-* | *-* | **** |
| *Agrococcus sediminis* strain LMR1168 | *+++* | *+* | *+++* | *-* | *-* | *-* | *-* | *-* | **** |
| *Agrococcus sediminis* strain LMR1169 | *+++* | *++* | *-* | *-* | *-* | *-* | *-* | *-* | **** |
| *Agrococcus sediminis* strain LMR1170 | *++* | *++* | *-* | *-* | *-* | *-* | *-* | *-* | **** |
| *Agrococcus* sp. LMR1171 | *++* | *+* | *-* | *-* | *+++* | *-* | *-* | *-* | ***** |
| *Bacillus altitudinis* strain LMR1175 | *++* | *++* | *-* | *-* | *-* | *++* | *-* | *+* | *1* |
| *Bacillus cereus* strain LMR1176 | *+++* | *++* | *+++* | *-* | *-* | *++* | *-* | *+* | *2* |
| *Bacillus cereus* strain LMR1177 | *+++* | *++* | *+* | *-* | *-* | *++* | *-* | *+* | *2* |
| *Bacillus cereus* strain LMR1178 | *+++* | *++* | *+* | *-* | *-* | *++* | *-* | *+* | *2* |
| *Bacillus paramycoides* strain LMR1180 | *+* | *++* | *-* | *-* | *+++* | *-* | *+++* | *-* | *1* |
| *Bacillus pumilus* strain LMR1181 | *-* | *++* | *-* | *-* | *++* | *++* | *-* | *+* | *1* |
| *Bacillus* *paramycoides* strain LMR1182 | *++* | *++* | *+++* | *-* | *+* | *+++* | *-* | *+* | *1* |
| *Peribacillus frigoritolerans* strain LMR1183 | *+++* | *++* | *+++* | *+* | *+* | *-* | *-* | *-* | *** |
| *Bacillus* *subtilis* strain LMR1184 | *++* | *++* | *+* | *-* | *-* | *+* | *-* | *-* | *1* |
| *Brevibacterium anseongense* strain LMR1185 | *++* | *++* | *+++* | *+++* | *+++* | *-* | *+* | *-* | **** |
| *Microbacterium resistens* strain LMR1188 | *+* | *++* | *++* | *+* | *-* | *-* | *-* | *-* | *2* |
| *Microbacterium resistens* strain LMR1189 | *+++* | *++* | *+++* | *-* | *-* | *+* | *-* | *-* | *2* |
| *Microbacterium resistens* strain LMR1190 | *+++* | *++* | *-* | *-* | *+* | *-* | *-* | *-* | *2* |
| *Microbacterium resistens* strain LMR1191 | *++* | *++* | *-* | *-* | *-* | *-* | *-* | *-* | *2* |
| *Microbacterium resistens* strain LMR1192 | *++* | *++* | *++* | *-* | *-* | *-* | *-* | *-* | *2* |
| *Microbacterium resistens* strain LMR1193 | *++* | *++* | *++* | *-* | *-* | *-* | *-* | *-* | *2* |
| *Microbacterium resistens* strain LMR1194 | *++* | *++* | *++* | *+* | *+* | *-* | *-* | *-* | *2* |
| *Microbacterium resistens* strain LMR1195 | *++* | *++* | *-* | *-* | *+* | *-* | *-* | *-* | *2* |
| *Microbacterium resistens* strain LMR1196 | *++* | *++* | *+* | *+++* | *+++* | *-* | *-* | *-* | *2* |
| *Microbacterium* *proteolyticum* strain LMR1197 | *++* | *++* | *++* | *+++* | *++* | *-* | *-* | *-* | *1* |
| *Neobacillus drentensis* strain LMR1179 | *++* | *++* | *++* | *-* | *+* | *-* | *-* | *-* | *1* |
| *Paenibacillus validus* strain LMR1198 | *+* | *+* | *-* | *-* | *+* | *-* | *-* | *-* | *1* |
| *Pseudarthrobacter* *oxydans* strain LMR1174 | *+* | *+* | *+* | *+* | *++* | *-* | *-* | *-* | *1* |
| *Pseudarthrobacter* *oxydans* strain LMR1200 | *++* | *++* | *++* | *+* | *+* | *-* | *-* | *-* | *1* |
| *Pseudarthrobacter* *phenanthrenivorans* strain LMR1203 | *+* | *+* | *+++* | *-* | *++* | *-* | *-* | *-* | *1* |
| *Pseudarthrobacter* *siccitolerans* strain LMR1204 | *+* | *++* | *-* | *-* | *+* | *-* | *-* | *-* | *1* |
| *Pseudarthrobacter* *oxydans* strain LMR1205 | *+++* | *++* | *+++* | *-* | *+++* | *-* | *-* | *-* | *1* |
| *Raoultella* *ornithinolytica* strain LMR1163 | *-* | *++* | *++* | *++* | *-* | *++* | *-* | *+* | *2* |
| *Stenotrophomonas rhizophila* strain LMR1208 | *++* | *++* | *++* | *-* | *-* | *-* | *-* | *-* | *1* |
| *Stenotrophomonas rhizophila* strain LMR1209 | *+* | *++* | *-* | *-* | *-* | *-* | *-* | *-* | *1* |
| Stenotrophomonas *rhizophila* strain LMR1210 | *-* | *-* | *-* | *-* | *++* | *++* | *-* | *-* | *1* |
| *Stenotrophomonas rhizophila* strain LMR1211 | *++* | *++* | *++* | *-* | *+++* | *++* | *-* | *-* | *1* |
| *Stenotrophomonas rhizophila* strain LMR1212 | *+* | *-* | *-* | *-* | *-* | *++* | *-* | *-* | *1* |

**: Parte, A. C., 2014. LPSN—list of prokaryotic names with standing in nomenclature. Nucleic acids research, 42(D1), D613-D616.*

***:* Brevibacterium anseongense *and* Agrococcus sediminis *have never been studied in vitro before.*

****: the risk group could not be known for unidentified species*


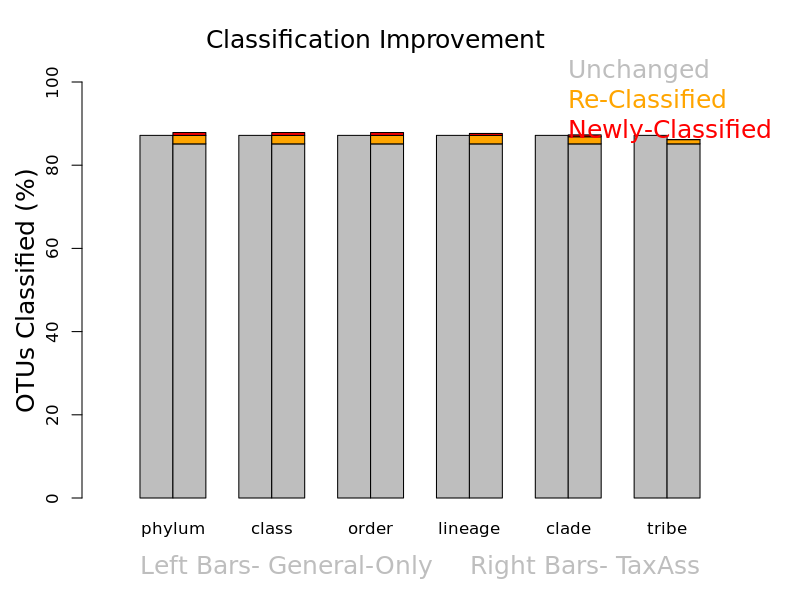


*Fig S1:* The TaxAss comparison between the culture-dependent and culture-independent sequences. The left bars indicate the SILVA-only classification, whereas the right bars represent the TaxAss classification, which incorporated both SILVA and a trainset containing the 41 isolated bacterial sequences in the present study. In red, we have the sequences that were classified by the trainset using TaxAss but were not classified when using only SILVA, in yellow we have the sequences that were classified by only SILVA but were re-classified by the trainset using TaxAss, while in gray, we have sequences that were classified similarly when using Silva and when using TaxAss.
